# Prevalence and Risk Factors of Mortality among Adult HIV Patients Initiating ART in Rural Setting of HIV Care and Treatment Services in North Western Tanzania: A Retrospective Cohort Study

**DOI:** 10.1155/2017/7075601

**Published:** 2017-06-15

**Authors:** Daniel Wilfred Gunda, Igembe Nkandala, Semvua Bukheti Kilonzo, Boniface Bartholomew Kilangi, Bonaventura Cornel Mpondo

**Affiliations:** ^1^Department of Medicine, Weill Bugando School of Medicine, 1464 Mwanza, Tanzania; ^2^Department of Medicine, Bugando Medical Centre, 1370 Mwanza, Tanzania; ^3^Department of Medicine, College of Health Sciences, University of Dodoma, 395 Dodoma, Tanzania

## Abstract

**Introduction:**

HIV still causes high mortality despite use of ART. This study was designed to determine the prevalence and risk factors of mortality among HIV patients receiving ART in northwestern rural Tanzania.

**Methods:**

A retrospective study of HIV patients on ART was done at Sengerema in Mwanza, Tanzania. The data on demography, date of HIV diagnosis, WHO stage, opportunistic infections, CD4, hemoglobin, ART regimen, and time and outcome on treatment as dead or alive were collected and analyzed using STATA version 11.

**Results:**

In total, 740 patients were studied. The median age was 35 (27–42) years with female predominance of 465 (62.8%). Of the participants, 261 (35.3%) had WHO stages 3 and 4 diseases. Most participants, 258 (34.9%), had baseline CD4 counts <200 cells/*μ*l. Deaths occurred in 86 (11.6%) patients which were independently associated with male gender (16.0% versus 9.0%, *p* = 0.015), being divorced (OR = 2.7, *p* < 0.001), WHO stages 3 and 4 (OR = 2.3, *p* = 0.05), CD4 <200 cells/*μ*l (OR = 3.4, *p* < 0.001), and severe anemia (OR = 6.6, *p* < 0.001).

**Conclusions:**

The mortality is high among HIV patients receiving ART in northwestern rural Tanzania. Universal testing could increase early diagnosis and treatment. A close follow-up of at-risk patients within the first year of ART could reduce the mortality of this subgroup of patients.

## 1. Introduction

HIV/AIDS is still an ongoing problem, especially in resource-limited countries, where it still causes high morbidity and mortality. Since its discovery in 1981, almost 78 million people have been infected with HIV [1] and as of today HIV has so far caused more than 34 million deaths [2]. HIV attacks CD4-positive cells as its host cells in the human body, reducing the number and quality of these immune cells, leading to increased morbidity and mortality from opportunistic infections [3]. By 2014, there were about 37 million people who were living with HIV/AIDS globally [4] with more than 70% of this global burden residing in countries in Sub-Saharan Africa (SSA) [4]. In Tanzania, about 1.4 million people are living with HIV/AIDS, representing 6% of SSA burden of HIV/AIDS and 4% of all people living with HIV/AIDS (PLWHA) globally [5].

Being one of the medical successes, the advent of antiretroviral therapy (ART) has dramatically improved the outcome of PLWHA [6, 7] as reflected in an overall reduction in morbidity and mortality from HIV-related opportunistic infections (OI) as a result of durable suppression of HIV replication and restoration of the body's ability to fight against OI [8]. Based on this, there is an ongoing worldwide expansion of HIV/AIDS care and treatment services with a goal line to improve the outcome of PLWHA and reduce the rate of new HIV infection. For instance, in 2015, more than 15 million of PLWHA had access to ART globally [9], and in 2011 more than 8 million of PLWHA had access to treatment services in SSA [10], while by 2013 more than 37% of PLWHA were already on ART in Tanzania [5].

These efforts on the other hand face a number of challenges including a proportion of patients who still die while receiving ART which ranges between 4.5 and 17% [11, 12] in most series. In Tanzania, the literature on the magnitude of this problem is still inadequate, especially in the rural setting of HIV/AIDS care and treatment services. This study therefore aimed at determining the prevalence and baseline risk factors of mortality among HIV patients receiving ART at care and treatment services in a rural setting of Tanzania.

## 2. Materials and Methods

### 2.1. Study Design and Study Setting

This was a retrospective cohort study which was conducted between July 2016 and February 2017. The study was done at Sengerema Designated District Hospital (DDH) care and treatment center (CTC) in Mwanza region. Sengerema DDH is one of the district level hospitals situated in the western part of Lake Victoria serving a catchment population of about 700,000 people with a bed capacity of about 300. Sengerema DDH runs both inpatients' and outpatients' activities on daily bases serving between 200 and 300 patients a day. CTC activities started way-back in 2008 as part and parcel of routine outpatients' services. The center provides both testing and treatment services, currently serving a total of more than 8000 patients, where 4000 are active on ARTs.

### 2.2. Study Population and Sample Size

This study involved all adult HIV-positive patients who were started on ART and continued with care and treatment services at Sengerema CTC between January 2008 and December 2015. A minimum sample size of 706 patients was estimated from Leslie Kish's formula (1965) for cross-sectional studies [13] assuming a mortality rate of 8% (ranging between 4.5 and 16.7%) among HIV patients receiving ART according to most studies [11, 12] with tolerable error of 0.03 at 95% confidence interval (CI).

### 2.3. Sampling Method and Data Collection

The sample was derived from a list of all patients who were diagnosed and started on ART at Sengerema CTC during the study period. Files of patients on ART were retrieved and a systematic sampling was done to get the required sample size. The skip interval “*K*” was obtained from the formula *K* = number of patients on ART/estimated sample size as used in other studies [14] and the starting point was randomly selected. When a file missed important information, the next file was taken instead. The selected files were then reviewed and information of research interest was extracted and recorded in a special tool, and data including age, sex, date of HIV diagnosis, WHO clinical stage, OIs, baseline CD4, complete blood count, year of ART initiation and regimen, and time and outcome on ART as dead or alive were collected and analyzed using STATA version 11.

### 2.4. Data Entry and Management

Data were computerized using EpiData version 3.1 and STATA version 11 (StataCorp LP, College Station, TX) was used for data analysis. Continuous variables were expressed as medians with interquartile range (IQR), while categorical variables were expressed as proportions with percentages. Advanced HIV was defined as CD4 count <200 cells/*μ*l (severe immune suppression) and/or presentation with WHO clinical stage 3 or 4 [14]. Hb level of 10–12/13 g/dL for females and males, respectively, was referred to as mild anemia, while moderate anemia and severe anemia were defined as Hb level of 8–10 g/dL and Hb < 8 g/dL, respectively, for both sexes [15, 16]. The proportion of patients who died in the course of their ART was calculated. The effect of different risk factors on the odds of mortality on ART was investigated. The odds ratio with 95% CI was calculated using univariate analysis followed by multivariate analysis model to assess the extent of association of different variables to the outcome of interest. In all our analysis, factors were said to be statistically significant when the *p* value was less than 0.05.

### 2.5. Ethical Clearance

The permission to conduct this study was obtained from the Catholic University of Health and Allied Sciences/Bugando Medical Center Joint Ethical Committee. The patients' files were handled by the researcher alone and the patients' identifiers including names and registration numbers were not included to maintain confidentiality.

## 3. Results

### 3.1. Demographic, Clinical, and Laboratory Characteristics of 740 Participants

**Figure 1:**
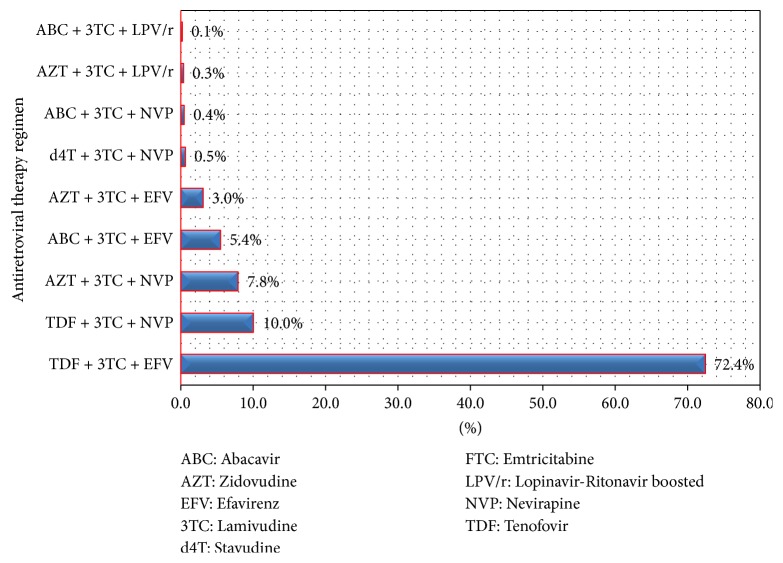
The distribution of combined antiretroviral regimen.

In this study, a total of 740 patients were included for analysis. The median age of the study participants was 35 (IQR: 27–42) years, with most patients (584 (78.9%)) being below 45 years. Female predominance of 465 (62.8%) was observed and 278 (37.6%) of participants were married. More than a third of participants, 261 (35.3%), had WHO stages 3 and 4 AIDS-defining illnesses with TB being the most common OI (197 (26.6%) at enrolment for HIV care and treatment services). The median baseline CD4 count was 256 (IQR: 132–469) cells/*μ*l, with more than one third of patients, 258 (34.9%), being enrolled with severe immune suppression with CD4 counts < 200 cells/*μ*l at baseline. About 288 (38.9%) of the study participants were anemic at enrolment with median Hb of 11.4 (IQR: 9.7–13.1) g/dL (Table 1) and the commonest ART regimen was a combination of Tenofovir, Lamivudine, and Efavirenz as summarized in Figure 1.

### 3.2. Prevalence and Risk Factors of Mortality among 740 Patients While Receiving ART

**Figure 2:**
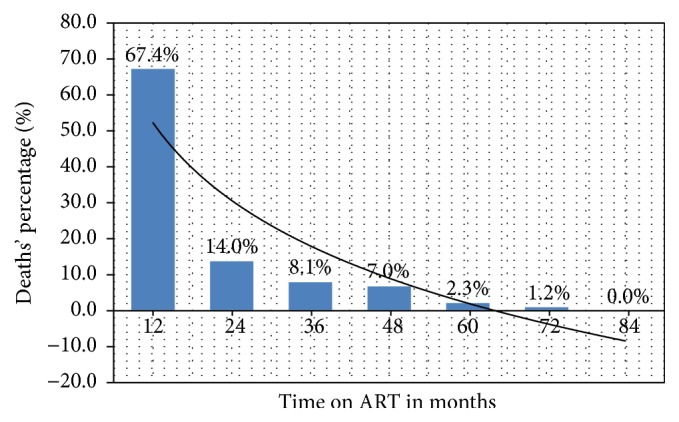
The distribution of deaths by months on ART.

In this study, 86 (11.6%) of the study participants died while receiving ART (Table 1). About 81.4% of all deaths occurred in the first two years of ART use with 67.4% and 14% occurring in the first year and the second year, respectively (Figure 2). The odds of mortality on receipt of ART were strongly associated with male gender (16% versus 9%, *p* = 0.015), being divorced (OR = 2.7, *p* < 0.001), WHO clinical stages 3 and 4 (OR = 2.3, *p* = 0.05), baseline CD4 counts of less than 200 cells/*μ*l (OR = 3.4, *p* < 0.001), and severe anemia on diagnosis of HIV (OR = 6.6, *p* < 0.001). The distribution of other factors was not statistically different (Table 2).

## 4. Discussion

The objective of this study was to determine the prevalence and assess the risk factors for death among adult HIV-positive patients on receipt of ART at a rural setting of HIV care and treatment service. In this study, 11.6% of patients died on receipt of ART, where most deaths (67.4%) occurred in the first year of treatment and the risk of death was independently associated with male gender, being divorced, WHO stages 3 and 4 AIDS-defining conditions, CD4 counts of less than 200 cells/*μ*l, and severe anemia at baseline.

These findings are comparable to several other studies. The mortality rate among HIV patients receiving ART in the index study is similar to prevalence of 11.7% reported from Senegal in 2006 [17]. But it is as well similar to that reported previously from Dar es Salaam by Mugusi et al. in 2012 [18]. In this study, the prevalence of death was 10.9% and 11.3% among those with HIV alone and among those with TB coinfection, respectively, suggesting that mortality on receipt of ART is still a common phenomenon among HIV patients attending both rural and urban settings of HIV care and treatment services in Tanzania.

Our mortality finding is higher than recently reported prevalence of 4.5% in Uganda in 2015 [12] and prevalence of 6.5% in a large cohort study from Europe and America in 2014 [19]. On the other hand, much higher prevalence of 16.6% and another prevalence of 29.7% were previously reported in Ethiopia [11] and northern Tanzania [20], respectively. These differences could probably be explained by differences in study settings. For instance, in European and American studies, HIV patients tend to be diagnosed and initiated on ART much earlier with potential less mortality on ART receipt. However, even with the present differences in mortality rate, the clinical message remains significantly similar that, despite the use of ART, the mortality among HIV-positive patients is comparatively higher than the general population in both developed and developing countries with comparable timing of these deaths in most series [21, 22]. For instance, in one study from Ethiopia, where 10.0% of patients on ART died in 6 years of follow-up, it was found that 62.9% of the reported deaths occurred in the first year of ART [23], while in a study by Johannessen et al. from Manyara, Tanzania, it was indicated that 72.7% of the reported deaths occurred within the first 12 months of ART initiation [20]. Similarly, in the index study, 67.4% of the deaths also occurred within the first year of ART, further underlying the observation that most of the deaths among HIV patients occur within the first year of ART initiation as a potential point of intervention. Observations suggest that most deaths in resource-limited settings are driven by advanced immune suppression characterized by low CD4 counts <200 cells/*μ*l [24].

Patients who start ART at baseline CD4 counts <200 cells/*μ*l have frequently been demonstrated to have a poor immune recovery [25, 26] with increased risk of both AIDS and non-AIDS-related morbidities and mortalities [27, 28]. A prior study from Uganda had indicated that deaths among HIV patients on ART in 3 years of follow-up were strongly related to specific diseases. In this study, 80 (14%) deaths occurred within one year of ART, where 69 (86%) of them were due to HIV-related causes including central nervous system infection like cryptococcal meningitis and toxoplasmosis (25%), active TB (16%), Kaposi sarcoma (10%),* Pneumocystis jiroveci* pneumonia (PJP) (7%), and severe anemia (7%); and only 8 (10%) were due to other medical conditions. Similar to our study, it was further shown that most of these deaths were among those patients who were severely immune suppressed at the time of ART initiation [24].

In agreement with other studies, a number of factors were found to independently augment the likelihood of mortality in the index study and therefore they could be useful clinically in planning strategies to optimize the overall outcome of HIV patients who are initiating ART in our setting. For instance, in this study, male patients were 1.8 times more likely to die while receiving ART than female counterparts. Similarly, in one study form Uganda, male patients were 1.37 more likely to die than female participants [12], while in Ethiopia male patients were 2.6 times more likely to die on receipt of ART [29]. This could partly be explained by the purported poor health seeking behavior among male patients as also reported previously [30], potentially leading to late HIV diagnosis and poor outcome on ART.

With this background, one study from China indicated that male patients were 1.73 times more likely to present with advanced HIV diagnosis than female patients [31], similar to a study from Nigeria, where male patients were 1.8 times more likely to present with advanced HIV diagnosis [32]. Also another study assessing mortality in Uganda had indicated that male patients were more severely ill with most of WHO clinical stages 3 and 4 AIDS-defining illnesses (36% versus 33%, *p* < 0.0001) and they had 37% more risk of death compared to female patients on receipt of ART (OR = 1.37, *p* < 0.001) [12]. Being linked to Prevention of Mother to Child Transmission (PMCT) services, studies suggest that female patients tend to be familiar with their HIV status much earlier and start on antiretroviral therapy promptly [33] with better outcome compared to their male counterparts. This poor outcome of male patients while receiving ART could as well be due to poor adherence to care and treatment services. Programs targeting male patients including male support or adherence groups in addition to universal HIV testing could potentiate early HIV diagnosis and treatment, thus improving the outcome of this subgroup of patients.

Furthermore, divorced patients had 2.7 times higher risk of dying on receipt of ART. Fewer studies have reported similar findings elsewhere. For instance, in one study from Uganda assessing gender-related mortality among HIV patients on ART, it was indicated that female patients who were either divorced, separated, or widowed were more likely to die on ART than male patients (48.0% versus 20.9%, *p* < 0.001) [33]. Another study from USA assessing marital status and HIV/AIDS mortality had similar observation that divorced and separated HIV patients had 4.3 times higher risk of mortality; however, this observation applied more for male patients [34]. This could probably be due to loss of a spouse as a potential treatment supporter.

Patients who were in WHO clinical stages 3 and 4 were also at increased risk of death on receipt of ART (2.3 times more likely than those with stages 1 and 2 diseases). Similar findings have been reported from several studies in other countries including Uganda (OR = 1.37, *p* < 0.001) [12] and Ethiopia (OR = 3.9, *p* = 0.002) [35]. In another study from Zambia, it was also indicated that patients in WHO clinical stages 3 and 4 had increased risk of mortality with adjusted hazard ratios of 1.8 and 2.9, respectively [36]. Severe immune suppression has frequently been shown to be associated with increased risk of fatal WHO clinical stages 3 and 4 AIDS-defining conditions including TB [37, 38]. Similar to several other studies [12, 35, 39], patients who were severely immune suppressed on diagnosis in the index study had 3.4 times higher risk of dying on ART than those who were less immune suppressed. In one study form Ethiopia by Setegn et al., patients who presented with TB on diagnosis of their HIV had 4.6 times higher risk of mortality on ART than those who were TB-negative on HIV diagnosis [29]. These findings are otherwise consistent with our observation in the index study that the risk of death was higher among patients who had TB compared to those who were TB-negative on diagnosis especially on a univariate analysis (OR = 2.8, *p* < 0.001). The underdiagnosis of TB in our setting could be the basis of loss of its association to the mortality on adjusted analysis. It has been shown that TB presents atypically with worsening immune suppression, frequently reducing the TB detection rate with increasing mortality from undetected cases of TB [40, 41]. In 2016, a follow-up autopsy study of severely immune suppressed patients in South Africa indicated that 47% of them had evidence of TB, where 88% of whom had EPTB [42]. Also studying the cause of deaths among HIV patients in resource-limited setting, a systematic meta-analysis study by Gupta et al. indicated that TB was the overall cause of death in 37.2% of the studied patients. In this review, it was further indicated that the prevalence rate of autopsy TB in Sub-Saharan Africa was 43.2% and some other settings had higher prevalence rate of more than 63% [43]. Since most of the modern methods for TB diagnosis including Gene Xpert and culture facilities are still relatively expensive and not readily available for routine use in our setting, TB awareness campaign and intensified frequent clinical screening of TB among HIV-positive patients coupled with high index of suspicion could increase the detection rate of TB, potentially improving the outcome of this subgroup of patients.

Severe anemia is another factor found to increase the risk of mortality on receipt of ART in the index study. Compared to other mild forms of the disease, severe anemia is known to have a mortality risk in this group of patients. In a EuroSIDA study assessing the effect of anemia in HIV progression, it was demonstrated that severely anemic patients had a high relative hazard of disease progression (7.1 versus 2.2) as compared to those with mild anemia and nonanemic individuals [44]. In assessing predictors of survival among HIV patients initiated on ART in Ethiopia, Mengesha and colleagues indicated that mortality increased with decreasing hemoglobin [45]. Also, in another study by Sullivan et al., a higher risk of death of 148% was demonstrated among HIV patients with moderate-to-severe anemia as compared to those with mild anemia or nonanemic patients [46]. We also had similar observation in this study, where the risk of mortality was 6.6 times higher among severely anemic patients as compared to those with less severe forms of anemia. The increased mortality among severely anemic patients in this study could partly be explained by cardiac complications that are mostly serious including heart failure and ischemic heart diseases, as it has also been documented previously [47]. On the other hand, the causes of anemia among these HIV patients could be severe and fatal including chronic renal failure [48] and disseminated opportunistic infections like tuberculosis [49], potentially increasing the mortality of HIV patients while receiving ART.

This study had a number of limitations. Being a single center-based study, the results from this study may not be generalizable. And being a retrospective study, some of vital information including compliance level could not be retrieved; missing data was also a common problem in the reviewed patients' records. Also since this was a retrospective study, the other limitations include lack of OIs diagnosis and no cause of death was recorded. The possibility that temporal changes may have occurred during the 7 years of the study such as changes of ART drugs and other guidelines like the screening practices for TB is also a limitation. However, our study assessed clients enrolled between 2008 and 2015 and enrolled 740 patients; we believe the sample size is bigger enough to study the desired outcomes. It is one of the few studies in Tanzania to assess the mortality rates and determine the associated factors in patients initiated on ART in current rural setting of HIV care and treatment.

In conclusion, the mortality is still high among HIV patients who are receiving ART in northwestern rural part of Tanzania. Most of the deaths occur within the first year following ART initiation. Male gender, severe anemia, being divorced, and advanced HIV at initiation of ART are significantly associated with mortality. Universal testing could serve as one of the strategies to insure early diagnosis and treatment of HIV before the disease is adversely advanced. Male support groups could improve the health seeking behavior among male patients. A close clinical follow-up of at-risk patients within the first year of ART could serve as a potential intervention to reduce the mortality of this subgroup of patients.

## Figures and Tables

**Table 1:** General demographic clinical and laboratory characteristics of 740 participants.

Variable	Frequency	Median (IQR) or percentage
*Age in years*	740	35 [27–42]
*Age group*		
≥45 years	156	21.1
<45 years	584	78.9
*Sex*		
Male	275	37.2
Female	465	62.8
*Marital status*		
Married	278	37.6
Divorced	160	21.6
Widow	154	20.8
Single	51	6.9
*WHO stage*		
3 & 4	261	35.3
1 & 2	479	64.7
*Baseline CD4 (cell/µl)*	740	256 [132–469]
*Baseline CD4 group*		
≤200 cell/*µ*l	258	34.9
>200 cell/*µ*l	482	65.1
*Hemoglobin (g/dL)*	740	11.4 [9.7–13.1]
Normal HB	452	61.1
Anemia total	288	38.9
Mild anemia	147	19.9
Moderate anemia	88	11.9
Severe anemia	53	7.2
*OI on diagnosis*		
Tuberculosis	197	26.6
Others	543	73.4
*Outcome on ART*		
Median time (Mo)	740	27 [17–37]
Dead	86	11.6
Alive	654	88.4

ART: antiretroviral therapy; CD4: cluster of differentiation 4; dl: deciliter; HB: hemoglobin; IQR: interquartile range; Mo: months; OI: opportunistic infection; WHO: World Health Organization.

**Table 2:** Univariate and multivariate analysis for factors associated with mortality among 740 participants while receiving ART.

Variables	Deaths while receiving ART		Unadjusted		Adjusted	
	No (*n* = 654)	Yes (*n* = 86)	OR (95% CI)	*p* value	OR (95% CI)	*p* value
*Sex*						
Male	231 (35.3)	44 (51.2)				
Female	423 (64.7)	42 (48.8)	1.9 (1.2–3.0)	0.005	1.8 (1.1–3.0)	**0.015**
*Age*						
≥45 years	141 (21.6)	15 (17.4)				
<45 years	513 (78.4)	71 (82.6)	0.8 (0.4–1.4)	0.380		
*Marital status*						
Married	248 (37.9)	35 (40.7)	1.2 (0.7–1.8)	0.618		
Divorced	129 (19.7)	31 (36.1)	2.3 (1.4–3.7)	0.001	2.7 (1.6–4.7)	**<0.001**
Widow	169 (25.8)	18 (20.9)	0.8 (0.4–1.3)	0.326		
Single	108 (16.6)	02 (02.3)	0.1 (0.0–0.5)	0.003		
*WHO stage*						
3 & 4	123 (32.6)	48 (55.8)				
1 & 2	441 (67.4)	38 (44.2)	2.6 (1.7–4.1)	<0.001	2.3 (1.0–5.5)	**0.05**
*Baseline TB*						
Yes	157 (24.1)	40 (46.5)				
No	497 (75.9)	46 (53.5)	2.8 (1.7–4.4)	<0.001	1.0 (0.4–2.5)	0.922
*Baseline CD4*						
<200 cells/*µ*l	208 (31.8)	50 (58.1)				
≥200 cells/*µ*l	446 (68.2)	36 (41.9)	3.3 (1.8–4.7)	<0.001	3.4 (2.0–5.7)	**<0.001**
*Anemia state*						
Mild anemia	138 (21.1)	09 (10.5)	0.4 (0.2–0.9)	0.023		
Mo anemia	078 (11.9)	10 (11.6)	1.0 (0.4–2.0)	0.936		
Sev anemia	030 (04.6)	23 (26.7)	7.6 (4.1–13.9)	<0.001	6.6 (3.4–12.9)	**<0.001**
*ART regimen*						
EFV based	532 (81.4)	65 (75.6)	0.3 (0.1–0.5)	<0.001		
LPV based	002 (00.3)	02 (02.3)	2.6 (0.3–24.0)	0.810		
NVP based	120 (18.3)	19 (22.1)	1.4 (0.8–2.3)	0.260		

ART: antiretroviral therapy; CI: confidence interval; EFV: Efavirenz; LPV: Lopinavir; Mo anemia: moderate anemia; NVP: Nevirapine; OR: odds ratio; Sev anemia: severe anemia; TB: tuberculosis; WHO: World Health Organization.

## References

[1] amfAR

[2] WHO

[3] Brooks J. T., Kaplan J. E., Holmes K. K., Benson C., Pau A., Masur H. (2009). HIV-associated opportunistic infections - Going, going, but not gone: The continued need for prevention and treatment guidelines. *Clinical Infectious Diseases*.

[4] WHO

[5] MOHSW

[6] Ka H. W., Chan K. C. W., Shui S. L. (2004). Delayed progression to death and to AIDS in a Hong Kong cohort of patients with advanced HIV type 1 disease during the era of highly active antiretroviral therapy. *Clinical Infectious Diseases*.

[7] Reniers G., Slaymaker E., Nakiyingi-Miiro J. (2014). Mortality trends in the era of antiretroviral therapy: Evidence from the network for analysing longitudinal population based HIV/AIDS data on Africa (ALPHA). *AIDS*.

[8] Le T., Wright E. J., Smith D. M. (2013). Enhanced CD4+ T-cell recovery with earlier HIV-1 antiretroviral therapy. *New England Journal of Medicine*.

[9] UNAIDS

[10] UNAIDS

[11] Jerene D., Endale A., Hailu Y., Lindtjøorn B. (2006). Predictors of early death in a cohort of Ethiopian patients treated with HAART. *BMC Infectious Diseases*.

[12] Rubaihayo J., Tumwesigye N. M., Konde-Lule J. (2015). Trends and predictors of mortality among HIV positive patients in the era of highly active antiretroviral therapy in Uganda. *Infectious Disease Reports*.

[13] Charan J., Biswas T. (2013). How to calculate sample size for different study designs in medical research?. *Indian Journal of Psychological Medicine*.

[14] Mhozya H., Bintabara D., Kibusi S., Neilson E., Mpondo B. C. (2015). Late-stage disease at presentation to an HIV clinic in eastern Tanzania: a retrospective cross-sectional study. *Malawi Medical Journal*.

[15] Gunda D. W., Kilonzo S. B., Mpondo B. C. (2016). Magnitude and correlates of moderate to severe anemia among adult HIV patients receiving first line HAART in northwestern Tanzania: A cross sectional clinic based study. *Pan African Medical Journal*.

[16] Meidani M., Rezaei F ., Maracy. M. R., Avijgan M., Tayeri K. (2012). Prevalence, severity, and related factors of anemia in HIV/AIDS patients. *J Res Med Sci*.

[17] Etard J.-F., Ndiaye I., Thierry-Mieg M. (2006). Mortality and causes of death in adults receiving highly active antiretroviral therapy in Senegal: a 7-year cohort study. *AIDS*.

[18] Mugusi S. F., Ngaimisi E., Janabi M. Y. (2012). Risk factors for mortality among HIV-positive patients with and without active tuberculosis in Dar es Salaam, Tanzania. *Antiviral Therapy*.

[19] Ingle S. M., May M. T., Gill M. J. (2014). Impact of risk factors for specific causes of death in the first and subsequent years of antiretroviral therapy among HIV-infected patients. *Clinical Infectious Diseases*.

[20] Johannessen A., Naman E., Ngowi B. J. (2008). Predictors of mortality in HIV-infected patients starting antiretroviral therapy in a rural hospital in Tanzania. *BMC Infectious Diseases*.

[21] Zwahlem M., Harris R., May M. (2009). Mortality of HIV-infected patients starting potent antiretroviral therapy: comparison with the general population in nine industrialized countries. *International Journal of Epidemiology*.

[22] Eyawo O., Franco-Villalobos C., Hull M. W. (2017). Changes in mortality rates and causes of death in a population-based cohort of persons living with and without HIV from 1996 to 2012. *BMC Infectious Diseases*.

[23] Tachbele E., Ameni G. (2016). Survival and predictors of mortality among human immunodeficiency virus patients on anti-retroviral treatment at Jinka Hospital, South Omo, Ethiopia: a six years retrospective cohort study. *Epidemiology and Health*.

[24] Castelnuovo B., Manaba Y. C., Kiragga A., Kamya M., Easterbrook P., Kambugu A. (2009). Cause-specific mortality and the contribution of immune reconstitution inflammatory syndrome in the first 3 years after antiretroviral therapy initiation in an urban African cohort. *Clinical Infectious Diseases*.

[25] Moore R. D., Keruly J. C. (2007). CD4+ cell count 6 years after commencement of highly active antiretroviral therapy in persons with sustained virologic suppression. *Clinical Infectious Diseases*.

[26] Hunt P. W., Deeks S. G., Rodriguez B. (2003). Continued CD4 cell count increases in HIV-infected adults experiencing 4 years of viral suppression on antiretroviral therapy. *AIDS*.

[27] Baker J. V., Peng G., Rapkin J. (2008). Poor initial CD4+ recovery with antiretroviral therapy prolongs immune depletion and increases risk for AIDS and non-AIDS diseases. *Journal of Acquired Immune Deficiency Syndromes*.

[28] Lichtenstein K. A., Armon C., Buchacz K. (2010). Low CD4^+^ T cell count is a risk factor for cardiovascular disease events in the HIV outpatient study. *Clinical Infectious Diseases*.

[29] Setegn T., Takele A., Gizaw T., Nigatu D., Haile D. (2015). Predictors of mortality among adult antiretroviral therapy users in southeastern Ethiopia: Retrospective cohort study. *AIDS Research and Treatment*.

[30] Nachega J. B., Hislop M., Dowdy D. W. (2006). Adherence to highly active antiretroviral therapy assessed by pharmacy claims predicts survival in HIV-infected South African adults. *Journal of Acquired Immune Deficiency Syndromes*.

[31] Jiang H., Yin J., Fan Y. (2015). Gender difference in advanced HIV disease and late presentation according to European consensus definitions. *Scientific Reports*.

[32] Agaba P. A., Meloni S. T., Sule H. M. (2014). Patients who present late to HIV care and associated risk factors in Nigeria. *HIV Medicine*.

[33] Alibhai A., Kipp W., Saunders L. D. (2010). Gender-related mortality for HIV-infected patients on highly active antiretroviral therapy (HAART) in rural Uganda. *International Journal of Women's Health*.

[34] Kposowa A. J. (2013). Marital status and HIV/AIDS mortality: Evidence from the US National Longitudinal Mortality Study. *International Journal of Infectious Diseases*.

[35] Biadgilign S., Reda A. A., Digaffe T. (2012). Predictors of mortality among HIV infected patients taking antiretroviral treatment in Ethiopia: a retrospective cohort study. *AIDS Research and Therapy*.

[36] Stringer J. S. A., Zulu I., Levy J. (2006). Rapid scale-up of antiretroviral therapy at primary care sites in Zambia: feasibility and early outcomes. *Journal of the American Medical Association*.

[37] Damtie D., Yismaw G., Woldeyohannes D., Anagaw B. (2013). Common opportunistic infections and their CD4 cell correlates among HIV-infected patients attending at antiretroviral therapy clinic of Gondar University Hospital, Northwest Ethiopia. *BMC Research Notes*.

[38] Kufa T., Mabuto T., Muchiri E. (2014). Incidence of HIV-associated tuberculosis among individuals taking combination antiretroviral therapy: A systematic review and meta-analysis. *PLoS ONE*.

[39] Utami S., Sawitri A. A., Wulandari L. P. (2017). Mortality among people living with HIV on antiretroviral treatment in Bali, Indonesia: incidence and predictors. *International Journal of STD & AIDS*.

[40] Sterling T. R., Pham P. A., Chaisson R. E. (2010). HIV infection-related tuberculosis: Clinical manifestations and treatment. *Clinical Infectious Diseases*.

[41] Chamie G., Luetkemeyer A., Walusimbi-Nanteza M. (2010). Significant variation in presentation of pulmonary tuberculosis across a high resolution of CD4 strata. *International Journal of Tuberculosis and Lung Disease*.

[42] Karat A. S., Omar T., Von Gottberg A. (2016). Autopsy prevalence of tuberculosis and other potentially treatable infections among adults with advanced HIV enrolled in out-patient care in South Africa. *PLoS ONE*.

[43] Gupta R. K., Lucas S. B., Fielding K. L., Lawn S. D. (2015). Prevalence of tuberculosis in post-mortem studies of HIV-infected adults and children in resource-limited settings: A systematic review and meta-analysis. *AIDS*.

[44] Lundgren J. D., Mocroft A., Gatell J. M. (2002). A clinically prognostic scoring system for patients receiving highly active antiretroviral therapy: Results from the EuroSIDA study. *Journal of Infectious Diseases*.

[45] Mengesha S., Belayihun B., Kumie A. (2014). Predictors of survival in HIV-infected patient after Initiation of HAART in Zewditu Memorial Hospital, Addis Ababa, Ethiopia. *International Scholarly Research Notices*.

[46] Sullivan P. S., Hanson D. L., Chu S. Y., Jones J. L., Ward J. W. (1998). Epidemiology of anemia in human immunodeficiency virus (HIV)-infected persons: results from the multistate adult and adolescent spectrum of HIV disease surveillance project. *Blood*.

[47] Groenveld H. F., Januzzi J. L., Damman K. (2008). Anemia and mortality in heart failure patients: a systematic review and meta-analysis. *Journal of the American College of Cardiology*.

[48] Fiseha T., Tamir Z., Seid A., Demsiss W. (2017). Prevalence of anemia in renal insufficiency among HIV infected patients initiating ART at a hospital in Northeast Ethiopia. *BMC Hematology*.

[49] Saathoff E., Villamor E., Mugusi F., Bosch R. J., Urassa W., Fawzi W. W. (2011). Anemia in adults with tuberculosis is associated with HIV and anthropometric status in Dar es Salaam, Tanzania. *International Journal of Tuberculosis and Lung Disease*.

